# Monitoring infection with *Piscine myocarditis virus* and development of cardiomyopathy syndrome in farmed Atlantic salmon (*Salmo salar *L.) in Norway

**DOI:** 10.1111/jfd.12974

**Published:** 2019-02-26

**Authors:** Julie Christine Svendsen, Stian Nylund, Anja B. Kristoffersen, Harald Takle, Julia Fossberg Buhaug, Britt Bang Jensen

**Affiliations:** ^1^ Norwegian Veterinary Institute Oslo Norway; ^2^ Pharmaq Analytiq Bergen Norway; ^3^ Marine Harvest Bergen Norway; ^4^ Lerøy Midt AS Sandstad Norway; ^5^ Present address: Cermaq Group AS Oslo Norway

**Keywords:** Atlantic salmon, cardiomyopathy syndrome, cohort study, epidemiology, field investigation, piscine myocarditis, *Piscine myocarditis virus*

## Abstract

An epidemiological study was carried out in Norway in 2015–2018, investigating the development of infection with *Piscine myocarditis virus *(PMCV) and development of cardiomyopathy syndrome (CMS) in farmed Atlantic salmon. Cohorts from 12 sites were followed and sampled every month or every other month from sea transfer to slaughter. PMCV was detected at all sites and in all sampled cages, and fish in six sites developed clinical CMS. The initial infection happened between 1 and 7 months post‐sea transfer, and the median time from infection with PMCV until outbreak of CMS was 6.5 months. Generally, fish from sites with CMS had higher viral titre and a higher prevalence of PMCV, compared to sites that did not develop clinical CMS. The virus persisted until the point of slaughter at most (11 out of 12) of the sites. The detection of PMCV in all sites suggests that PMCV is more widespread than previously known. Screening for PMCV as a tool to monitor impending outbreaks of CMS must be supported by observations of the health status of the fish and risk factors for development of disease.

## 1 INTRODUCTION

Cardiomyopathy syndrome (CMS) is a disease that affects farmed Atlantic salmon (*Salmo salar* L.). It is one of the most serious diseases in Norwegian aquaculture, resulting in reduced fish welfare, increased mortality and substantial economic loss for the industry (Brun, Poppe, Skrudland, & Jarp, 2003). The disease was first detected in Norway in the 1980s, then of unknown aetiology (Amin & Trasti, 1988). Since then, the disease has made its appearance in the Faroe Islands (Poppe & Sande, 1994; Poppe & Seierstad, 2003), Scotland (Rodger & Turnbull, 2000) and Ireland (Rodger, Mccleary, & Ruane, 2014). CMS has typically been known to affect fish in the second year of the grow‐out phase. However, there is an increasing number of field reports of disease on younger fish, and scientific reports describe the disease occurring on fish groups five or six months post‐sea transfer (Fritsvold et al., 2015; Wiik‐Nielsen, Alarcón, Jensen, Haugland, & Mikalsen, 2016). Histological findings include inflammatory changes primarily in the spongious part of the atrium and ventricle, whilst the compact myocardium usually remains unaffected (Bruno, Noguera, & Poppe, 2013; Ferguson, Poppe, & Speare, 1990). In severe cases, the changes can lead to a rupture of the cardiac wall, with a rapid, fatal outcome (Ferguson et al., 1990).

Cardiomyopathy syndrome was shown to transmit horizontally between fish in 2009 (Bruno & Noguera, 2009; Fritsvold et al., 2009), and the causative agent was identified in the two subsequent years, when a novel, totilike virus named *Piscine myocarditis virus *(PMCV) was detected and linked to the disease (Haugland et al., 2011; Løvoll et al., 2010). PMCV is a naked, double‐stranded RNA virus with a relatively small genome. It differs from other members of the *Totiviridae*—family in several ways, amongst them through its choice of a vertebrate host and its manner of extracellular transmission (Haugland et al., 2011). Studies using RT‐PCR analysis have shown that there is a good correlation between levels of virus found in heart, and the severity of cardiac lesions (Haugland et al., 2011; Timmerhaus et al., 2011). Little is known about the biophysical properties of PMCV, and it has proven difficult to grow in cell culture. The latter has made the development of a vaccine challenging, and as of now, there is none on the market.

Cardiomyopathy syndrome is not a notifiable disease, neither in Norway nor for the World Organization for Animal Health (OIE), and there is no official control programme for CMS in Norway. As such, the exact number of annual cases is uncertain. The Norwegian Veterinary Institute has diagnosed CMS in approximately 100 seasites every year since 2013, and additional cases are identified at other laboratories (Hjeltnes, Jensen, Borno, Haukaas, & Walde, 2018). On affected sites, the disease may manifest itself acutely with an abrupt increase in mortality, or as a more prolonged phase with moderate losses over time.

Diagnosis of CMS is based on histopathology. PCR for PMCV is suggestive of CMS, but finding of the virus alone without any pathological findings does not support a diagnosis of CMS.

In the years following the detection of PMCV, several studies have investigated the epidemiology of the disease, including other transmission pathways, reservoirs and risk factors for disease. Vertical transmission of CMS is suspected and is a focus of current research. Two separate studies have detected PMCV in heart samples from brood fish as well as in roe and milt, fertilized eggs and yolksac fry (Jensen, 2017; Wiik‐Nielsen, Ski, Aunsmo, & Løvoll, 2012). There are no known occurrences of clinical disease in the freshwater phase. Findings indicate that the most important, known reservoir for the virus is farmed salmon. An overview of known and investigated sources is presented in the review recently published by Garseth and colleagues (Garseth, Fritsvold, Svendsen, Bang Jensen, & Mikalsen, 2018).

In a study of risk factors for CMS in Norway, the median time from sea transfer to a CMS diagnosis was shown to be 16 months (Jensen, Brun, Fineid, Larssen, & Kristoffersen, 2013). The same study concluded that the likelihood of developing disease increased with length of time in the grow‐out phase, infectious pressure from neighbouring sites, as well as CMS in previous generations (Jensen et al., 2013). However, there is a lack of knowledge of when fish cohorts are infected, how infection is related to clinical outbreaks and for how long the infection persists.

The main aim of this study was to gain more knowledge about time of infection with PMCV, length of the interval between infection with PMCV and clinical outbreak of CMS, and how long the virus persists in the population after infection. An additional aim was to use findings to discuss whether screening for PMCV can be used as a predictor for outbreaks of CMS.

## 2 MATERIALS AND METHODS

### 2.1 Study population

**Figure 1:**
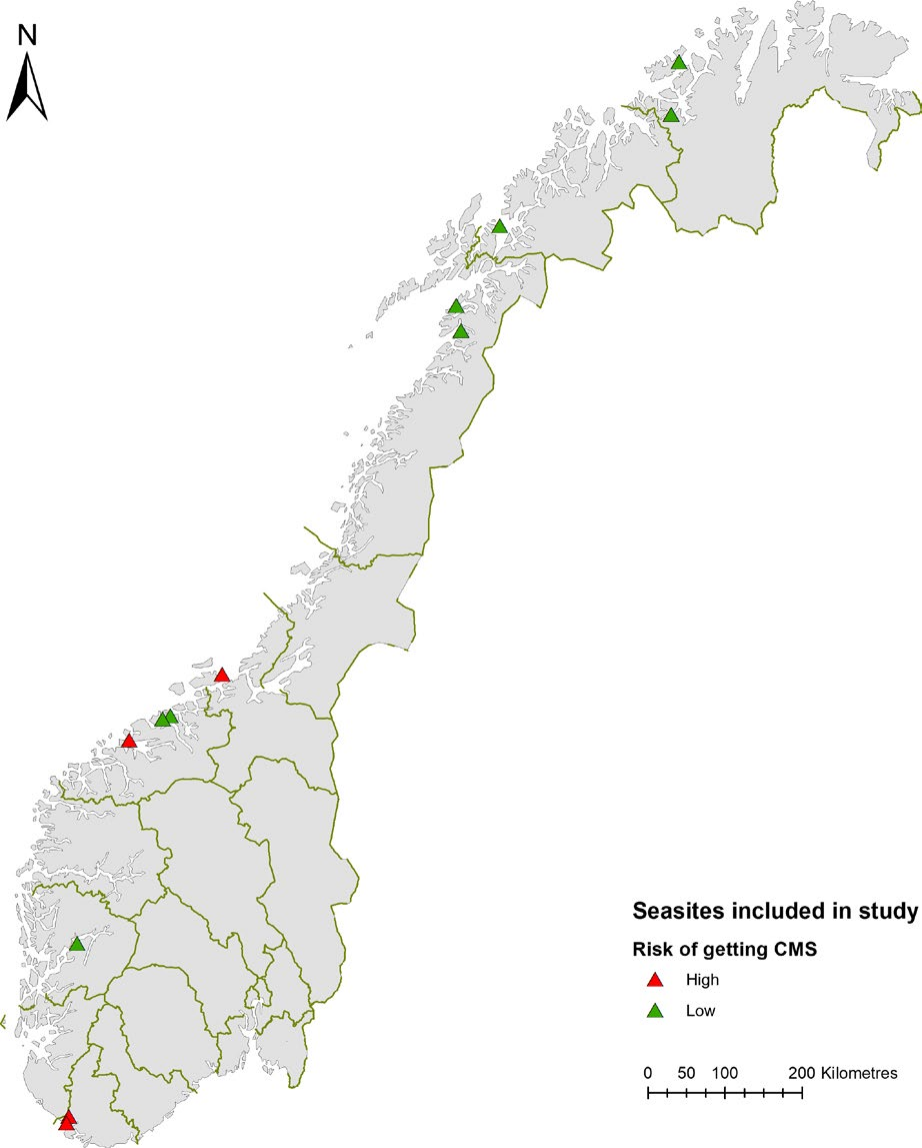
Map of Norway showing the position of the 12 seasites included in the study. Red triangle indicates that the seasite is defined as a high‐CMS risk site, green triangle indicates that the seasite is defined as a low‐CMS risk site

The monitored population was farmed Atlantic salmon, stocked on seasites along the Norwegian coastline (Figure 1). Three sites were stocked in the fall of 2015, four were stocked in the spring of 2016 and five in the fall of 2016. Four fish farming companies participated in the study, and the fish originated from different hatcheries. In this study, a cohort is defined as a group of fish in a separate cage, originating from the same hatchery and stocked at the same time. Two cohorts were followed on each site, with the exception of one site where three cohorts were followed. The farmers themselves made the selection of sites and cohorts to follow.

### 2.2 Study design

Groups of Atlantic salmon were followed throughout the marine grow‐out phase in a prospective, longitudinal cohort study. Selected sites were defined as high‐, or low risk by the farmers, based on previous CMS history (Figure 1). Sampling for PMCV‐PCR (as described below) was done approximately every month on high‐risk sites, and every other month on low‐risk sites. Approximately 30 individuals were sampled from each cage on every sampling occasion, that is 60 fish from each site.^1^ This included both recently deceased and moribund fish, as well as individuals with no external clinical signs of disease. Sampling personnel were instructed to collect 20 recently deceased and 10 live fish per cage, if possible.

Upon suspicion of clinical disease, an extended sampling was performed. The purpose of this was to investigate the spread of the virus within sites, as well as to be able to make a disease diagnosis. In addition to collecting samples from the cages already included in the study, 60 fish from each of two additional cages (i.e., 120 extra fish) were sampled on these occasions.^2^ Tissue samples for histopathological assessments were also collected. The decision on whether to perform an extended sampling was based on development in cycle threshold (Ct) levels and prevalence of PMCV, together with information from the fish health service regarding mortality and the general health status on the sites. On two additional sites where clinical disease was suspected, tissue samples for histological assessment and RT‐PCR analysis were collected only from the cages that were followed throughout the study due to time limitations. These are not defined as extended sampling events.

A site was categorized as CMS‐positive based on results from two laboratory tests, that is RT‐PCR and histopathology. The start date for an outbreak was set as the date of the sampling which rendered the positive test results.

### 2.3 Tissue sampling

All samples were collected either by fish health personnel (fish health veterinarians or biologists) or by site workers given practical education on sample collection by the fish health service.

Live fish were captured by convenience sampling, whilst recently deceased fish were selected from the accumulated dead fish pool at the bottom of the net pen.

Prior to tissue sampling, all living fish were euthanized either by an overdose of anaesthetic or by a blow to the head. Samples were collected from the tip of the ventricle, in a size measuring approx. 2 mm*2 mm*2 mm. These were then stored on RNA‐later and shipped for RT‐PCR analysis performed by Pharmaq Analytiq. If possible, samples were shipped overnight on ice. If kept longer, samples were refrigerated for the first 24 hr and then stored in a frozen state until they were shipped.

For histopathological assessment, tissue samples measuring approx. 1 cm*1 cm*0.4 cm were collected from the heart, spleen, liver, head kidney, pancreas and skeletal muscle and fixed in 10% buffered phosphate formalin.

### 2.4 Laboratory methods

#### 2.4.1 RNA extraction

Tissue samples were processed using a Qiagen's Universal Biorobot, with the compatible RNA purification kit (RNeasy 96 Universal Tissue Kit), according to the manufacturer's recommendation. Extracted total RNA was eluted in a final volume of 100 µl of the supplied kit elution buffer.

#### 2.4.2 Real‐time RT‐PCR

Extracted RNA from tissues of salmon was tested by Taqman real‐time RT‐PCR (qScript XLT‐1 1‐Step RT‐ qPCR ToughMix, QUANTAbio). During the real‐time RT‐PCR screening a house‐keeping gene, elongation factor 1 alpha (EF1A), was used as an internal control (Olsvik, Lie, Jordal, Nilsen, & Hordvik, 2005), and a specific assay was used for detection of PMCV (Nylund et al., 2018). The primer and probe concentrations had been optimized and found to be 900 nM for all primers used and 225 nM for the corresponding probes. The samples were run in simplex for the internal control and triplicates for detection of PMCV in standard 384‐well plates. All assays were run in a total volume of 10 µl in each well, with 2.5 µl of isolated total RNA as the template. Plates were analysed in an Applied Biosystems 7900 HT real‐time machine under standard conditions. Each run consisted of 40 cycles, and the samples were considered positive when the fluorescence signal increased above a set threshold of 0.09. The PMCV assay has a repeatable Ct at 34.7. This level was set based on 10 replicates of ten‐fold dilutions of a DNA stock and denotes the mean Ct‐value of the highest dilution for which the 10 replicates were positive. Although Ct‐values above this threshold are possible, they are likely to not be reproducible under the same reaction conditions. A standard curve was generated using a 10‐fold serial dilution of DNA in three parallels. Regression analysis, standard curve slopes *s* of Ct versus log quantity DNA and amplification efficiency *E* where *E *= [10^1/(‐slope)^] −1 were calculated. The coefficient of determination, *R* ^2^, was 0.99. The slope for the assay was −3.44, and the amplification efficiency *E* was 0.95. For the EF1Ab assay, the corresponding values were 0.99, −3.98 and 0.78.

The formalin‐fixated samples were processed, embedded in paraffin, sectioned 4–5 μm thin and stained with haematoxylin and eosin in accordance with routine procedures. Sections were scanned at 400x and scored in accordance with Table 1 in Wessel et al. (2018).

**Table 1:** Information about the participating sites

Site number	Company	Region	Risk of CMS	Generation	No of. sampling events	Time of slaughter
1	A	North	Low	Spring 16	7	Jan 18
2	A	North	Low	Spring 16	8	Feb 18
3	A	North	Low	Spring 16	10	Oct/Dec 17
4	A	North	Low	Fall 16	9	April 18
5	B	North	Low	Fall 16	7	April 18
6	B	Mid	High	Spring 16	12	Aug/Sept 17
7	C	Mid	Low	Fall 16	6	Oct 17
8	C	Mid	Low	Fall 16	7	Nov 17
9	C	Mid	High	Fall 16	16	March 18
10	D	South	High	Fall 15	10	Dec 16
11	D	South	Low	Fall 15	5	May 17
12	D	South	High	Fall 15	12	Dec 16

Note

Risk of cardiomyopathy syndrome (CMS) is based on the farmers experience, as described in the text.

### 2.5 Data collection and analysis

The field samples were collected from October 2015 to April 2018. The screening results for all individual sampling events were compiled and sorted into categories in Microsoft Excel (version 14.4.4). This included Ct‐values, date of sampling, cage number and comments regarding health status of the individual fish. Based on the latter, all samples where categorized into four categories with regard to health status of the fish: “Normal” means that the fish did not display any external signs of disease; “Dead/moribund” means a recently deceased or gravely ill individual of otherwise normal size and condition; “Loser” refers to thin, weak individuals typically suffering from long term effects of prior diseases such as PD, or other ongoing challenges affecting growth and general health status; and,“unknown” indicates that we do not know the health status of the fish, that is this has not been commented upon sampling. Further details about the individual sites on geographical location, risk category, hatchery origin, time of stocking, number of cages on the sites, generation (fall or spring) and eventually time of slaughter were collected from the participating companies. The general information about the sites is summarized in Table 1.

Descriptive data analysis was carried out using the statistical software Stata (version 13.1), and a statistical comparison between the categories of health status was performed using the Wilcoxon test in R (version 3.4.4).

## 3 RESULTS

### 3.1 Onset of infection and disease

**Figure 2:**
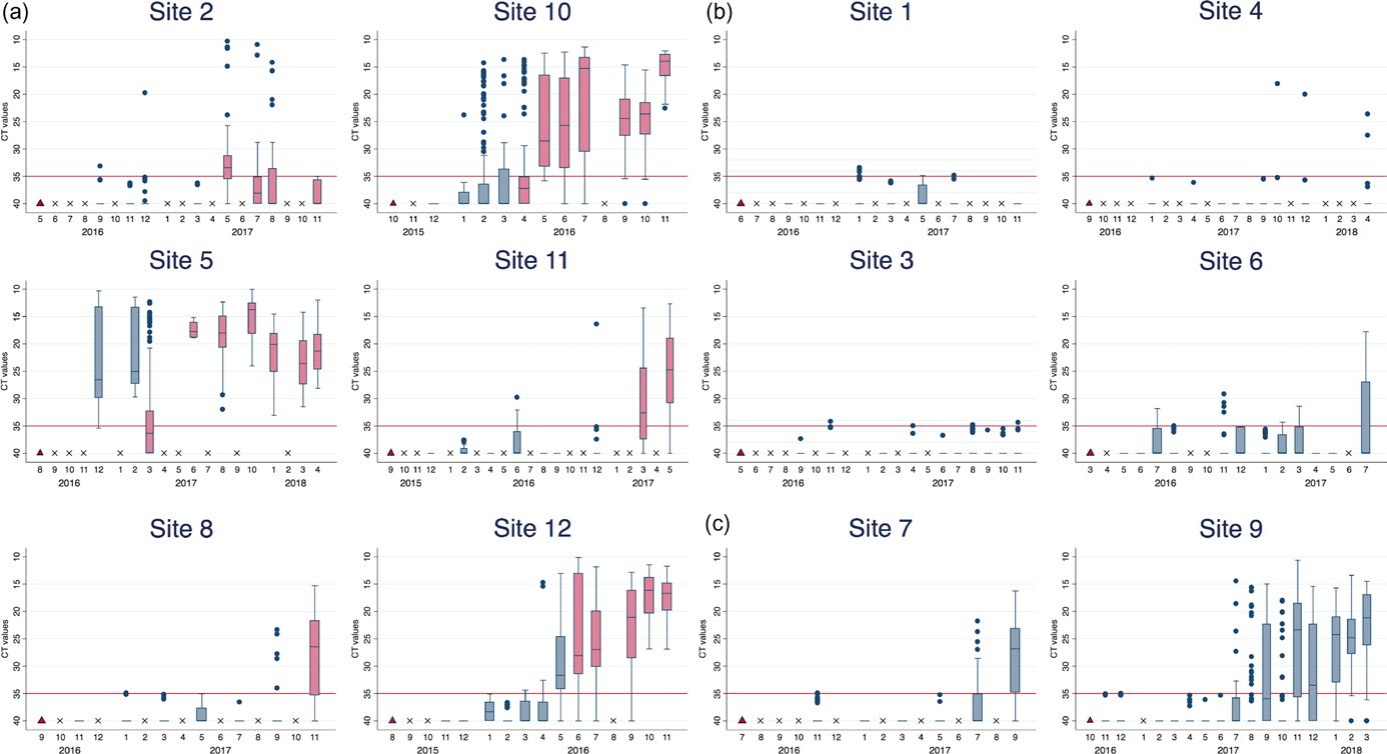
Boxplots displaying the development of PMCV in individual cohorts. The box represents the interquartile range (IQR) where the middle 50% of the data are contained. The line in the box illustrates the median, representing the middle of the dataset (50th percentile). Whiskers are drawn from the upper and lower quartile, ±1.5 IQR. Outliers mark points beyond either whisker. The red line indicates the cut‐off point (35) that is commercially used for the RT‐qPCR test. The triangle marks the month when the fish were stocked at the site, and an x denotes a month with no sampling. The first red box marks the time of the CMS diagnosis, and boxes remain red for the rest of the time at sea. (a) Development in CT levels on sites with clinical CMS. (b) Development in CT levels on sites without clinical CMS. (c) Development in CT levels on sites with suspicion of CMS

Viral RNA was detected through RT‐qPCR on all the participating sites and in all sampled cages. Fish in 6 of the 12 monitored sites developed clinical CMS (Figure 2a), whilst fish in four of the sites did not develop CMS (Figure 2b). On two sites, there was an increase in viral prevalence and a drop in Ct levels; however, a CMS diagnosis could not be made based on histopathology (Figure 2c). These sites were defined as suspected clinical cases.

The time of primary detection of PMCV varied from 1 to 4 months post‐sea transfer in the two CMS suspect sites, whilst the median time was 4 months in both the sites that did not develop CMS and in those that did (Table 2). Further, the median time from sea transfer to first PMCV detection was 4 months for both spring locations and fall locations. Overall, the time from sea transfer to primary detection of PMCV varied from 1 to 7 months post‐sea transfer, between the 12 sites. There was no difference in the time from sea transfer to PMCV infection between the three geographical regions (north, mid and south).

**Table 2:** Time (in months) from sea transfer until PMCV infection and from infection until development of clinical disease

Disease status	Months from sea transfer → PMCV (+) (median (range))	Months from PMCV (+) → CMS (+) (median (range))	Months from sea transfer → CMS (+) (median (range))
CMS‐positive (*n* = 6)	4 (3–5)	6.5 (3–13)	11 (6–18)
CMS negative (*n* = 4)	4 (4–7)	n/a	n/a
CMS suspect (*n* = 2)	2.5 (1–4)	n/a	n/a

Note

CMS: cardiomyopathy syndrome; PMCV: *Piscine myocarditis virus*.

The time from sea transfer until clinical disease in the CMS‐positive sites ranged from 6 to 18 months. The median time from PMCV infection to clinical outbreak was 6.5 months, with a range of 3–13 months (Table 2).

There was a higher proportion of sites with clinical disease amongst the fall sites (5/8), compared to the spring sites (1/4). Both the suspected clinical sites had fall fish.

Extended samplings were performed on seven sites (sites 1, 2, 5, 6, 7, 10 and 11; Figure 2), between 4 and 18 months post‐sea transfer. On the background of this extended sampling, four of the seven sites were classified as CMS‐positive, two remained CMS negative, and one was CMS suspect. PMCV was detected in fish from all additionally sampled cages, including those on sites that remained CMS negative. On two additional sites (site 8 and 12), samples of fish were collected for RT‐PCR and histopathological assessment only from the two cages followed throughout the study. Results classified both sites as CMS‐positive.

### 3.2 Development of infection and disease

On the six sites with clinical CMS, PMCV was detected after 3–5 months at sea. On site 2, 8, 11 and 12, the detection of PMCV was followed by some months with high Ct‐values, before a marked decrease in Ct value corresponding to increase in viral load (Figure 2a). The CMS diagnosis was made either concurrent with or shortly after this increase, and the viral loads remained elevated for the rest of the production cycle.

On the four sites without clinical CMS, the image is quite different: PMCV was detected but remained at generally low levels. Viral RNA was detected on three of the sites until harvest. On site 1, there was no detection of PMCV at the final sampling; however, the prevalence of PMCV in preceding sampling events was so low that a negative result does not exclude viral presence (Figure 2b).

In the two sites where CMS was suspected, but not confirmed, the infection patterns were very similar to that of the sites which developed clinical CMS (Figure 2c).

At the first detection of PMCV, the prevalence varied from 2% to 55%, with the majority of detections having high Ct‐values, corresponding to low viral loads. On the last sampling before slaughter, the prevalence was highest on the CMS‐positive (33.3%–100%) and CMS suspect (82% and 90%) sites. In the CMS negative sites, the prevalence ranged from 0% to 46.7%. On this last sampling event, the Ct‐values were predominantly below 35 in the CMS‐positive and CMS suspect sites, but still high in the CMS negative sites.

### 3.3 Ct‐values by state of fish

**Figure 3:**
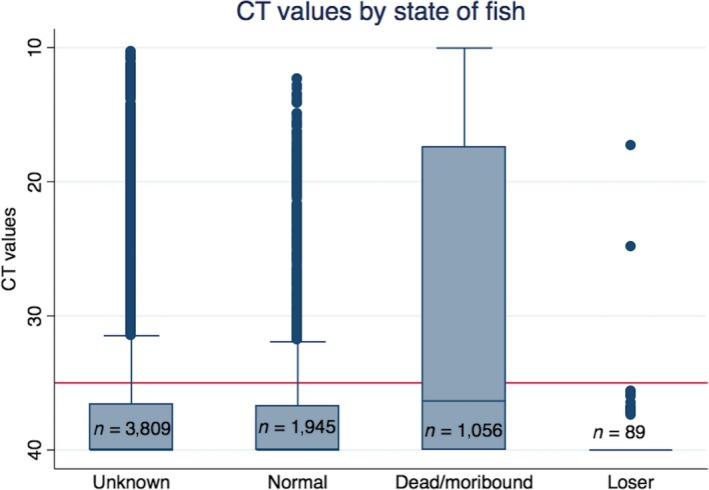
Comparison of CT‐values from sampled fish of different health status. Results shown in boxplots as explained for Figure 2. For explanation of the state of fish, see text under Data collection and analysis

When comparing Ct‐values from all sampled fish categorized into groups of “unknown,””Normal,” “Dead/moribund” and “Loser,” the group with “Dead/moribund” fish generally had significantly lower Ct‐values (*p* < 0.001; Figure 3). The compiled results do not meet the desire in the design of the study (with 20 recently deceased and 10 live individuals per sampling event). This is both due to practical challenges in the field, where sampling personnel had to deviate from the plan, as well as missing notations describing the health status of the fish.

## 4 DISCUSSION

*Piscine myocarditis virus* was detected at all sites and in all sampled cages at different time points of the grow‐out phase, and fish on six sites developed clinical CMS. There was a higher proportion of sites with clinical CMS (five of eight) amongst the fall‐stocked sites compared to the spring‐stocked sites (one of four). However, three of the four spring‐stocked sites were in the North, where the prevalence of CMS is lower, so it is possible that the occurrence of CMS had nothing to do with stocking time, but rather geography. Fish from sites with CMS had overall lower Ct‐values, and the prevalence was higher, compared to sites without the diagnosis. Dead or moribund fish generally had lower Ct‐values compared to live fish with no external clinical signs. The virus persisted until the point of slaughter at most (11 out of 12) of the sites.

At the initialization of the study, the farmers had categorized the farms into either high‐CMS risk or low‐CMS risk, based on previous experiences with CMS. The low‐CMS risk farms were not expected to be neither infected with PMCV nor to develop CMS. This was especially true for the farms in northern Norway, where the prevalence of CMS historically has been low (Hjeltnes et al., 2018). However, we found that PMCV was present in all sites, also in the northern region. Furthermore, four of the six sites that developed CMS were low‐risk sites, according to the farmers. This suggests that there is a disagreement between the perceived risk of CMS and the true risk. It also suggests that CMS is overlooked and/or underreported, since it is likely that the reason it was detected in the present study was because we were actively looking for it.

Several of the CMS‐positive sites follow the same pattern with initial detections of PMCV with high Ct‐values, which persists for a few months, until a sudden increase in prevalence and a decrease in Ct‐values happens concurrent with an outbreak of CMS. However, on site 2 the Ct levels did not undergo a marked development. On this site, a CMS diagnosis was made in May in 2017, and the fish remained in sea for another half year. It was reported that the disease took a clinically mild form on the site. This site was situated in northern Norway, an area in which reports of CMS have historically been low, although increasing over the past two years (Hjeltnes et al., 2018). It was also stocked with spring fish. Both are factors that could direct to explain why some sites experience severe disease and great losses, whilst others seem to make it through with much less severe consequences.

At the first PMCV detection, there was some variation in prevalence between the sites, but in general, the initial prevalence was low. Quite a lot of the detections had Ct‐values above the diagnostic cut‐off value. This means that on a regular screening report, they would be regarded as negative results. Values above the diagnostic cut‐off value have been included in the data set to ensure coverage of the early stages of infection, where virus levels were expected to be low, even though they are considered to be non‐reproducible.

At the last sampling before harvest, the picture was quite different. At all the CMS‐positive sites, the prevalence of PMCV was very high, except at the one with a less severe outbreak. On the CMS‐positive sites, the general image is also dominated by low Ct‐values. This was also the case for the two sites with suspected disease. The CMS negative sites, however, generally had a lower prevalence and the Ct‐values were higher. These results concur with the previous consensus of there being a good correlation between virus levels and clinical disease (Haugland et al., 2011; Timmerhaus et al., 2011).

The virus seems to be widespread and has been found on four sites where there was no subsequent development of clinical CMS. This indicates that the virus may exist in populations without ensuing clinical disease. Detecting PMCV does not necessarily indicate an impending disease outbreak, and the low prevalence and viral loads does not make it a suitable tool for early prediction of disease. However, screening can be purposeful to get an overview of the greater picture on a site. A development with increasing prevalence, as well as elevated viral loads, may be a warning signal. Used together with fish health assessments, this can form a basis for the decision to implement preventative measures.

In the protocol for sampling, we requested samples from 20 recently diseased fish supplied with 10 healthy fish, in order to ensure that the fish most likely to have infection or clinical disease were sampled. However, the compiled results revealed that only about 1/3 of the fish where the health status was known were dead or moribund. This could be because there sometimes simply was not as many as 20 dead fish at the time of sampling. In more than half of the samples, the state of fish was not listed when the samples were submitted. But when comparing Ct‐values from the Dead/moribund with the healthy fish, Ct‐values were lowest in the “Dead/moribund” group, indicating that this could be the best group to target when screening for the virus. Thus, we might have been able to detect infection at an earlier time or amongst a larger part of the fish if a greater part of the samples had been from dead or moribund fish. Another aspect of the sampling is the choice of sampling material. Studies have shown that PMCV is primarily found in areas affected with lesions (Wiik‐Nielsen, Lovoll et al., 2012). The lesions are believed to develop sequentially first in the atrium and subsequently in the ventricle (Haugland et al., 2011). Therefore, it might be assumed that the best tissue to sample for early detection of PMCV would be the atrium. For screening purposes, however, it is often more practical to use the tip of the ventricle, since this is easier to sample, especially for personnel who might not be so experienced in sampling and have little time to do so. In this project, we have used the protocol recommended by Pharmaq Analytiq who offers analysis of screening samples for PMCV for commercial use, and thus, we believe that this reflects well how the results of screening would be in real life.

The detection of PMCV in all sampled cages in this study could indicate either that the virus spreads efficiently between cages on a site or that there is an element of vertical transmission. The study was not designed to look at the transmission patterns between cages. It is known from other viral diseases, and also from reports from the field, that there can be major differences with regards to prevalence and clinical signs between cages in a site. The cages to be followed within the present study were chosen by the farmers, and in most cases, they chose cages with smolt from different suppliers, since they had a personal interest in finding out if there was a difference in susceptibility to infection between different smolt groups. In order to gain a thorough understanding of factors that influence disease transmission, a study including production data from more than 1,500 fish groups has been performed concurrent with this one by the authors, and the results will be published soon.

In another study performed as part of the present project, we have looked at vertical transmission as a possible additional route of infection. The results indicate that this could indeed be a possible transmission pathway under the current production systems (Jensen, 2017). A support for this is the recent findings of CMS in the Faroe Islands which have been linked to introduction with eggs imported from Norway (Garseth et al., 2018), and a reportedly high prevalence of PMCV amongst broodfish. In our study, we have also found PMCV early after sea transfer in several sites, further suggesting that they could have been carrying the infection from the freshwater phase.

In the present study, the median time from sea transfer to clinical CMS was 10 months, which is remarkably less than the 17 months that has previously been reported (Jensen et al., 2013). This gives support to the reports of a shift in the occurrence of CMS towards younger fish. However, some of the diagnoses in the study might have been forced earlier, as there was an increased awareness of CMS on participating sites.

The median time from infection with PMCV to clinical outbreaks of CMS was four months, but with a large range from 3 to 10 months. This makes it difficult to understand transmission pathways, when investigating reasons for clinical outbreaks. In the only published study on risk factors for CMS, infection pressure based on the number of neighbouring sites with CMS adjusted by seaway distance was found to be an important risk factor for development of CMS (Jensen et al., 2013). In that study, there was no information on the actual occurrence of PMCV, and only sites with clinical CMS were included in the calculations of infection pressure. Since infection pressure was found to be significant anyway, this could indicate that shedding of sufficient amounts of virus to cause transmission of disease only happens from fish that are clinically affected by infection. Thus, an option for control is to try and mitigate disease outbreaks, even if virus has been detected. This will of course also be beneficial for the farms already infected.

## CONFLICT OF INTEREST

Author Stian Nylund is affiliated with Pharmaq Analytiq which offers screening for salmon viruses included PMCV.

## NOTES

1 Ninety fish from the site where three cohorts were followed.

2 Except on two sites, one where 30 fish were collected from each of the two additional cages, and one where 60 fish were collected from one additional cage only.
